# Misclassification of HIV infection status among serologically diverse populations in Nigeria: implications for test and treat program

**DOI:** 10.1186/s12913-025-12617-9

**Published:** 2025-03-26

**Authors:** George I.E. Eluwa, Scott Geibel, Steven Callens, Lung Vu, Vincent J. Wong, Isa Iyortim

**Affiliations:** 1 Diadem Consults Initiative, Abuja, Nigeria; 2 Population Council, Washington DC, USA; 3 Faculty of Medicine and Health Sciences, Ghent University, Ghent, Belgium; 4 World Bank, Washington DC, USA; 5 USAID Office of HIV/AIDS, Washington D.C., USA; 6 United States Agency for International Development, Abuja, Nigeria

**Keywords:** HIV misclassification, HIV positive, Nigeria, Key populations

## Abstract

**Background:**

In 2015, the World Health Organization (WHO) launched the Test and Treat policy which supports antiretroviral treatment for all people with HIV, irrespective of CD4 count or clinical stage. This was adopted in 2016 in Nigeria. This policy resulted in scaleup of HIV testing strategies and differentiated models of care including community-based ART. This study evaluated the HIV testing algorithm and assessed the rates of misclassification of HIV status among newly diagnosed clients.

**Methods:**

Between February and August 2018, whole blood samples were collected from clients newly diagnosed with HIV in Lagos and Benue states. HIV status wasconfirmed with rapid tests using the serial algorithm during outreach sessions for both key populations and general populations. HIV positivity was confirmed using GenScreen™ HIV1/2.O Antibody only ELISA test (BioRad, USA). Optical density (OD) for each sample was measured with the use of Emax microplate reader set at endpoint 450 wavelength. Based on manufacturer’s algorithm, sample OD and calculated cut-off value ratio, an OD < 1.0 was interpreted as negative and > 1.0, positive. Concordance between rapid test algorithm result and ELISA was used to estimate the proportion of samples that were misclassified.

**Results:**

A total of 788 samples were collected from newly diagnosed clients across 4 sites in Lagos and 3 sites in Benue. Samples were collected from 212 and 178 key populations (KPs) clients in Lagos and Benue, respectively, and from 206 and 192 general population (GPs) clients in Lagos and Benue, respectively. Mean OD was 3.75 (IQR:3.70–3.81) with a standard deviation of 0.13. There was a 100% concordance between rapid test and ELISA results and no misclassification identified.

**Conclusion:**

We identified no instances of misclassification of positive HIV status suggesting that all clients who have been placed on treatment truly had HIV infection. The 100% concordance rate recorded from all the sites may be attributable to the maturity of the HIV program in Nigerian with a concomitant standard quality assurance system for both clinical and outreach testing services. This finding supports the implementation of the Test and Treat policy that Nigeria has adopted. Scale up of Test and Treat and community ART is thus recommended to increase access to treatment.

## Introduction

HIV testing is the entry point into HIV care and treatment and a diagnosis of HIV infection requires life-altering changes and decisions. Three policies thrust provide the momentum for rapid expansion of HIV testing services (HTS) globally. These include (i) the Joint United Nations Program on HIV and AIDS (UNAIDS) fast track targets which aims to ensure that 90% and 95% of people living with HIV know their status by the year 2020 and 2030 respectively [1]; (ii) the World Health Organization’s (WHO) policy on *Test and Start* which endorsed treatment initiation irrespective of clinical and immunological stage [2] and (iii) WHO’s policy on differentiated care that promotes community- based models for ART delivery [3].

The availability of rapid HIV diagnostic tests (RDTs) has enabled national country HIV programs to achieve this expansion and implement relevant treatment policies [4, 5]. The WHO algorithm guides the development of national testing algorithms, and it recommends different algorithms for populations with high (> 5%) or low (< 5%) HIV prevalence [6, 7]. WHO prequalified RDTs are required to have a diagnostic and clinical sensitivity of 99% and a specificity of 98% and when used correctly have been reported to have a diagnostic accuracy comparable to that of Enzyme Linked Immunosorbent Assay (ELISA) [6, 8–10], however studies have shown that the RDTs are still vulnerable to misdiagnosing HIV [4, 11, 12].

Misdiagnosis of HIV is defined as the incorrect reported outcome of an HIV test, i.e. incorrectly classifying a test result as HIV positive when it is truly negative and vice versa, thus misdiagnosis could be false positive or false negative [5]. Studies have reported both classes of misdiagnosis and various reasons have been cited as causes of misdiagnosis. This includes limitations of the RDTs, operator-related factors (e.g. clerical mix-ups, interpretation of weak banding), facility-related factors such as poor storage of reagents, inappropriate testing strategies/algorithms, high volume of clients leading to overburdened providers or low client volumes that result in poor provider proficiency, substandard training and lack of supportive supervision and sub-optimal national testing algorithms [4, 5, 13]. Cross reactivity, whereby an antibody binds to an antigen that differs from its originator antigen has also been reported to be a cause of false positive HIV misdiagnosis [4, 14–20].

WHO recommends the parallel or serial algorithm for HIV testing with different algorithms for high and low HIV prevalence populations. In high prevalence populations, the WHO algorithm requires two sequentially tested RDTs to be positive at first trial or at second trial following a repeat of first two tests if there is a discordance between the first and second test while in low prevalence populations a positive result is reported if two sequentially tested RDTs are positive at first trial or three RDTs are positive at second trial if there is a discordant result [6]. In addition, for quality assurance, WHO recommends retesting for verification at ART initiation [6]. The 2016 Nigerian National Guidelines for HIV Prevention, Treatment and Care endorses both parallel and serial testing, however serial testing is recommended for routine HIV testing and discordant results and resolved with a tiebreaker without repeat of the first two RDTs [21]. In 2016, Nigeria adopted the Test and Start policy which required that clients identified positive should be initiated on treatment irrespective of their CD4 count. However, since the adoption of the Test and Start policy, there has been no evaluation to determine if clients tested outside non-clinical settings are assigned the correct HIV status. This study evaluated HIV positive misclassification among high HIV prevalence population (men who have sex with men and female sex workers) and low prevalence population (general population). Findings from the study have implications for the test and start program in Nigeria.

### Nigeria’s HIV testing quality assurance program

Organizations that provide HIV testing services (HTS) are guided by the national quality assurance program, which is coordinated by the national technical working group (TWG) on HIV testing services. This TWG ensures that the 12 quality essentials recommended by WHO are delivered during HTS training for counsellor testers. These 12 quality essentials constitute the fulcrum of the quality management system implemented at community and facility levels in Nigeria. These quality essentials include registration of all implementing partners providing HTS with the state ministry of health, training and certification of HIV counsellors and testers, controlled sourcing/purchase and distribution of rapid test kits, standardized management of data capture tools (client intake forms, daily test kit usage), quarterly review of first line discordance rate (Determine versus Unigold), external proficiency testing, annual rapid test kits validation, and performance monitoring of the national algorithm.

## Methods

### Setting

This study is nested within a larger study that assessed the effect of treatment models on test and start treatment and community antiretroviral treatment among key populations in Nigeria. The study was conducted in Lagos and Benue states located in south west and north central Nigeria respectively. In both locations, from men who have sex with men (MSM), female sex workers (FSW) and the general population (GP) samples were collected from newly diagnosed clients seen at outreaches events until desired sample size of 700 samples was attained. HIV testing at the outreaches was done by trained counsellor-testers. Table 1 shows the heterogeneity of HIV prevalence among the study population.

**Table 1:** HIV prevalence disaggregated by population and state

Type of Population	Lagos (%)	Benue (%)
Men who have sex with men^a^	32.3	NA
Female sex workers brothel-based^a^	7.5	36.5
Female sex workers non brothel-based^a^	8.4	14.2
General population^b^	1.4	5.3

*NA* Data not available for MSM in Benue state. ^a^Data obtained from Integrated Biological and Behavioural Surveillance Survey, 2013, Federal Ministry of Health. ^b^Data obtained from National AIDS Indicator and Impact Survey, 2018. Federal Ministry of Health

### Operational definition

A highly sensitive test accurately detects true positive among those with the disease, and thus fewer cases of disease are missed. The specificity of a test is its ability to designate an individual who does not have a disease as negative. A highly specific test means that there are few false positive results [22]. Concordance is defined as the measure of agreement between measurements and refers to the degree of agreement between two (or more) sets of measurements [23]. A gold standard test refers to a diagnostic method with the best accuracy to identify true positives or true negatives [22].

### HIV testing

In Nigeria, a serial algorithm is recommended by the Federal Ministry of Health and RDTs approved for use by the guideline include Determine, Unigold, Statpak, Double-check Gold and Sure-Check, based on a previous assessment conducted in 2007 [21]. A second phase field evaluation of the testing algorithm was conducted in 2012, and the current recommended serial algorithm is as follows: first line, Determine™ HIV-1/2 (Abbott Laboratories); confirmation of positive, Uni-Gold™ HIV (Trinity Biotech) and tie-breaker, HIV 1/2 Stat-Pak^®^ Assay (Chembio Diagnostics Systems, Inc.). The sensitivity (ability to detect true positives) of the three rapid tests range between 98 − 100% while for GenScreen ELISA, sensitivity is 100% [24].

### Sample size and recruitment

For assessment of misclassification, based on a systematic review that reported global false positive rates ranging from 0 to 45% [5], and a Nigeria study that estimated false positive rates of 0% (95% CI: 0.00 to 1.59) in low prevalence and 1.16 (95% CI: 0.21 to 6.30) in high prevalence settings [25], we conservatively anticipated a misclassification rate in Nigeria of approximately 2%. Based on this and with an absolute precision of ± 1%, we estimated a sample size of 700. A purposive sample of 350 MSM and FSWs and 350 GPs who were newly diagnosed HIV positive by RDTs were recruited into the study and had blood samples collected by a lab scientist at the community. MSM were eligible if they were at least 16 years of age, reported engaging in anal intercourse with another man in the past six months, newly diagnosed HIV positive and willing to provide consent. Similarly, FSWs were eligible if they were 16 years of age, sold sex for at least once in the past six months, newly diagnosed HIV positive and willing to provide consent. For the GPs, they were eligible if they were 16 years of age, newly diagnosed HIV positive and willing to provide consent. All respondents provided consent prior to collection of biological samples following their rapid test.

### Laboratory procedure

#### Choice of validation HIV tests

HIV antibody re-testing was performed according to the national rapid testing algorithm in the laboratory. Serum samples confirmed as double-positive on RDTs by the laboratory were tested using ELISA (Genscreen^®^ HIV 1/HIV 2 Version 2, BioRad, Netherlands). Genscreen^®^ HIV 1/HIV 2 Version 2 has been used as a gold standard reference test for Nigeria’s 2007 and 2012 evaluations of rapid testing kits [26].

#### Specimen acquisition and processing

Ten millilitres (10mls) of venous blood were drawn from the antecubital vein of every participant into plain vacutainer tube after pre-test counselling. Two (2) capillary tubes were filled, and the remaining blood volume labelled, allowed to stand for two hours to retract into serum. For biorepository, samples were spotted onto filter paper for dry blood spot (DBS), while the two capillary tubes were used for HIV antibody re-testing according to the site algorithm. Serum was transferred into a transparent plain 2.0 ml vial plastic tube for ELISA test confirmation.

#### Transportation and storage

Serum samples were dispatched in cold-chain boxes daily by road and stored in referral laboratory until all samples had been collected for the study. They were subsequently batched and transported to the reference laboratory for confirmatory ELISA test.

#### Specimen analysis

Serum samples collected in a 2.0 ml micro vial were stored in −80 C to maintain sample integrity. Genscreen™ ULTRA HIV Ag-Ab-72,386 (Lot: 8E0122) was used for confirmatory analysis. Two medical laboratory scientists that were competent in quality management system (QMS) and ELISA conducted the assay, interpreted the results, and documented results appropriately. Testing protocol as described in the manufacturers’ manual was followed. Optical density (OD) for each sample was measured using Emax microplate reader set at endpoint 450 wavelength. Based on manufacturer’s interpretation algorithm, sample OD and calculated cut-off value ratio, < 1.0 was interpreted as negative and ratio > 1.0 was interpreted as positive.

## Results

A total of 788 confirmatory blood samples were collected from newly diagnosed HIV-positive clients representing key and general populations across three sites in Lagos state and two sites in Benue state. This comprised 212 samples and 178 samples from Lagos and Benue states, respectively for KPs yielding a total of 390 samples. For the general population, a total of 206 samples and 192 samples were collected for Lagos and Benue states, respectively yielding a total of 398 samples. Among MSM median age was 24 years (interquartile range {IQR} 21–27) and 30 years (IQR: 26–34) in Lagos and Benue states respectively. Among FSWs, median age was 31 years (IQR: 25–38) and 29 years (IQR: 25–33) in Lagos and Benue states respectively. Except for FSWs in Benue state (85%), less than 50% of the MSM in Lagos and Benue, FSWs in Lagos and GPs in Benue and Lagos who offered samples had received an HIV test in the previous three months prior to the study.

For the 788 samples assayed, the Median OD was 3.75 (IQR:3.70–3.81) with a standard deviation of 0.13. For the MSM, FSWs and GPs samples, there was 100% concordance between rapid testing algorithm result and ELISA results. There was no misclassification identified across any of the samples assayed either for MSM, FSWs or GPs.

## Discussion

This is the first study in Nigeria that has assessed misclassification of HIV positives among populations with high and low HIV prevalence (KP and GP) from different community settings, and the perfect concordance between rapid test algorithm result and confirmatory ELISA test bodes well for the country’s fledging test and start program.

False-positive HIV tests have been reported widely for both RDTs and ELISA [18, 27–41], and a key cause of this has been attributed to cross reactivity among RDTs. A study in Democratic Republic of Congo reported that 50% of all false positive cases for RDTs were based on gp41 reaction, a common antigen used by many RDT tests [18]. However, this cross reactivity is restricted to RDTs and not between RDTs and ELISA and implies that the perfect concordance reported in this study is attributable to other factors.

The level of concordance reported in our study is not a new finding in a resource limited setting. Shanks et al. assessed levels of false positives in three countries by confirming with Western Blot or Orgenics Immunocomb Confirm^®^ HIV Confirmation test and reported zero false positives among forty-three samples tested [4]. The testing algorithm was serial, similar to that used in the Nigerian national HIV testing program which includes tie breaker. The WHO HIV testing guidelines recommend a minimum of two sequential positive RDTs in high prevalence settings and three positive RDTs in low prevalence settings; neither of these algorithms includes a tie breaker [6]. The tie-breaker algorithm, wherea positive result following one positive result and one negative result is interpreted as confirmed positive result, has been reported to be highly susceptible to false results caused by cross-reactivity [42]. A study in Ethiopia that used a tie-breaker algorithm reported a false positive rate of 7.7% despite the use of RDTs with high specificity [42]. The Rakai cohort study in Uganda, in which a tie-breaker algorithm of Determine/Stat-Pak/Unigold RDTs was used in a setting with HIV prevalence of 11.2%, reported a false positive proportion of 43.7% [28, 43]. Another study in Uganda among a higher prevalence population reported a higher proportion of false positives (48.2%) with the use of tiebreakers, however confirmation was by DNA PCR [8, 43]. Boeras et al. [44] conducted large studies in Zambia and Rwanda on the tiebreaker algorithm and reported the proportion of false positives to be 46% [43].

This study used the national algorithm which involved at the time of the study– the use of a tiebreaker test—and is still being used in Nigeria at the time of this writing.Furthermore, while most of the studies cited above were conducted under laboratory conditions, over 50% of the samples were collected during outreach seasons in the communities in which the settings are more prone to error and a strong HTS quality system limits HTS errors. Despite the use of the tie-breaker algorithm testing strategy—which has led to sub-optimal results in multiple other studies—the integration of additional quality assurance systems may have contributed to correct test results in our population sample.

Johnson et al. conducted a systematic review on the misdiagnosis of HIV and reported that factors related to poor management and supervision were the most common reasons cited for poor quality HIV test [5]. While twenty-one studies reported user error (errors performing RDTs, wrong interpretation of results, misapplication of buffer, inaccurate reading time) as causes of poor quality HIV test, twenty studies reported poor management and supervision (work load stress, lack of training, poor adherence to testing strategies and management of supplies) and this included user errors. Poor participation in external quality assessment schemes (EQA), poor site level supervision and testing performed by under-trained or ineligible staff were also cited as poor management related causes [5]. An EQA scheme in Nigeria reported that over 40% of laboratory respondents had not being formally trained on HIV testing and only 33% of the sites used an approved national testing algorithm [45]. A study in South Africa that assessed 38 health facilities, reported low compliance to standard operating procedures and only 3.4% of HIV tests were conducted correctly [5]. The Nigerian national HIV testing program has a robust quality assurance system with rigorous training, retraining and certification of providers, controlled sourcing/purchase and distribution of RDTs to partners, quarterly review meeting of first line discordance rate, internal and external proficiency testing, use of standardized tools to capture usage of RDTs and a cap of number of tests to be performed per day by a provider. These quality assurance activities have strengthened the program management and supervision for HTS in Nigeria and our zero discordance may possibly be attributable to this high level of supervision.

Our study has some limitations. Weakly reactive tests which may arise from human error or reagent variability have been associated with false positive results [5, 15, 18], with some studies reporting 100% of weak positive results being false positives. Our study did not capture which tests were weakly reactive; however, all tests reacted positively with the confirmation test. Our target included both KPs and GPs to achieve the sample size. While HIV prevalence is higher among KPs compared to GPs, identifying new positives remains a challenge. The use of community based organizations facilitated the identification of KPs enrolled into the study. The application of WHO algorithm to high or low prevalence populations was not assessed as this study aligned with the national algorithm that recommends serial testing with a tiebreaker for discordant test. However, the diverse populations both high and low HIV prevalence groups assessed in this study provides a robust assessment of the HTS program and in addition the inclusion of results obtained from less optimal outreach settings further strengthens the application of this result to HIV program in Nigeria. Future studies comparing other testing algorithms are recommended.

## Conclusion

This 100% concordance between HIV positive results obtained from both high HIV and low HIV prevalence groups is evidence that providers in the national program are highly trained and adhere to standard operating guidelines. However, while the quality assurance program of the national program has strengthened the overall quality of the HTS program, the concordance reported is in the absence of a three assay (A1/A2/A3) result under the new WHO HIV testing guidelines [46]. As Nigeria continues to scale up HIV testing using different strategies in order to meet the UNAIDS 95:95:95 and to support the national test and start policy, programs must continue to adhere to the high level of standards with continuous technical oversight from the national program. While the quality assurance efforts used in the national program and by extension in this study appear to have supported good diagnostic outcomes, there is a need to update national algorithms to meet WHO standards.

## Data Availability

Data is available upon request.
